# The First Hundred Years of the Scientific Field of Anaesthesiology and Reanimation in the Republic of Turkey

**DOI:** 10.4274/TJAR.2023.231387

**Published:** 2023-12-27

**Authors:** Hatice Türe, Haluk Gümüş

**Affiliations:** 1 Yeditepe University Faculty of Medicine, Department of Anaesthesiology and Reanimation, İstanbul, Turkey; 2 Retired, Anaesthesiology and Reanimation Specialist, Ankara, Turkey

**Keywords:** Anaesthesiology and reanimation, history, the Republic of Turkey

## Abstract

As a scientific field, anaesthesiology and reanimation, with their significant place in the medical structure, have been practised since the beginning of surgical procedures. Today anaesthesiology and reanimation speciality cover more complex techniques and areas than alleviating patients’ pain during surgery. In the first hundred years since the proclamation of the Turkish Republic, the path covered in our scientific field is to pave the way for the next hundred years.

**Main Point**

• In the first hundred years since the proclamation of the Turkish Republic, the path covered in our scientific field is to pave the way for the next hundred years.

## Introduction

The scientific field of anaesthesiology and reanimation, with scientific and technological innovations, has been improved from the first definition of anaesthesia, which is “painlessness and numbness required for surgical procedure”, and developed into an indispensable branch that manages the perioperative medical status of the patient by balancing it's vital functions as a whole and by maintaining that balance. In Intensive Care Units, part of the anaesthesiology and reanimation field, patients needing advanced diagnoses and follow-ups are treated. Algology, a relatively new science, covers all pain management therapies in pain clinics and is growing with recent conformational changes.

The improvement and progress made with the guidance of pioneer physicians of our field in the first hundred years of the Turkish Republic will pave the way and enlighten us in future centuries.

### Main Text

Since the first day of human history, anaesthesia has been required to perform surgery.^1^ The modern history of our area has been shaped by the energy of our young and dynamic Turkish Republic, surviving the Independence War. In the first years of the Turkish Republic, while the Turkish people coming out of war were creating the new future, physicians loyal to their country made intense efforts in the field of anaesthesia. “Cemil Topuzlu, Besim Ömer Akalın, Orhan Abdi Kurtaran, Rıza Nur, Burhanettin Toker, Kâzım İsmail Gürkan, Akif Şakir Sakar, Ahmed Asım Onur, and Halit Ziya Konuralp” contributed to anaesthesiology and reanimation field, seeing its indispensability in performance of surgical procedures.^2,3,4,5,6^

When written sources about these periods are reviewed from the articles published by Burhanettin Toker in 1924, Kâzım İsmail Gürkan in 1926, and H. Ziya Konuralp in 1931, we learn that newly developed drugs were used widely in our country. Just before university reforms of 1933 as modern and scientific developments applied according to global standards, the articles “Painless Labor” by Tevfik Remzi Kazancıgil in 1929, “Monograph of Painless Labor” by Asim Onur in 1932, two articles about painless labour, and “Pernokton in Labor Analgesia” by Ziya Üstün in 1933, are essential documents of the history of anaesthesia. One of the pioneer anaesthesiology and reanimation specialists in Turkey, Prof. Dr. Cemalettin Öner, states that he has encountered 26 articles from this period.^1,3^ As anaesthesia and reanimation were a novelty field worldwide, new devices were being developed to anaesthetise patients. Following the university reform in Turkey, in 1937, famous surgeon, Prof. Dr. Rudolph Nissen brought the first anaesthesia device to Istanbul University Medical School to the I. Surgical Ward. However, at that time, no one volunteered to use this device.

Despite the increasing economic and political crises in the world during the Second World War (1935-1945), scientific and industrial revolutions have been initiated in the young Republic of Turkey to reach to the level of developed countries. At that time, ss a milestone, an anaesthesia device was brought to Istanbul University Medical School by request of Prof. Dr. Burhanettin Toker (1948). As a surgical resident, Dr. Sadi Sun has been assigned to use that mentioned device. In 1949, another milestone for anaesthesiology and reanimation field, the first endotracheal intubation has been performed by Burhaneddin Toker and Sadi Sun.^5,6,7,8,9,10,11,12,13,14,15^ In 1950, Dr. İhsan Günalp, Dr. Ali Yücel, Dr Cemil Aksoy, Dr. Hüsrev Polat and Dr. Orhan Bumin started performing endotracheal intubation in hospitals also in Ankara.

With the transition from one-party period to multiple-party period, the country’s political and geopolitical agenda became more active, and although the country remained neutral until the final stages of World War II, it always remained on the agenda due to its political position. Parallel the world direction, until the 1950s, the anaesthesia and reanimation field was not a separate branch but performed by physicians practicing in surgical fields or by assistant health providers under surgeons. In this period, Dr. Simon Batmaz, Dr. Melih Erhan and Dr. Hüseyin Ergönenç must be mentioned as pioneers of this field, as they were the first physicians to provide anaesthesia.^5,7,8,9,15^  In 1950, Prof. Dr. Burhanettin Toker requested that the anaesthesiology speciality be a separate branch in our country, with an application letter to then Minister of Health Ekrem Hayri Üstündağ. As the application of anaesthesia increased during surgery and more complicated procedures were performed, the first anaesthesia specialist was assigned to Haydarpasa Numune Hospital in 1953. Surgeon Dr. Hüsnü Öztürk was given as a “surgical and anaesthesia specialist” by the Ministry of Health due to his interest and experience from his European studies. During the same period, Prof. Dr. Robert Kucher, from Wien, was invited to establish an anaesthesia clinic at the same hospital. His successor Dr. Wolfgang Wirtinger, from Wien, with a decree dated 3^rd^ January 1955 and numbered 3239, established the first anaesthesia clinic and officially started anaesthesia training. Therefore 1955 is the first official establishment of our branch. Dr. Cemalettin Öner became the first official anaesthesia trainee of the Ministry of Health.^5,6,7,8,9,10,11,12^ In addition to his identity as an academician, Dr. Öner contributed also made great strides in the field of anaesthesia and intensive care in our country structurally.

As women, whose education was ignored during the Ottoman era, started being an active part of educational and work life during the Republician period. For example, women were given the right to vote and be elected in Turkey in 1934, long before many European countries. The first female anaesthesiologists of the new and contemporary Turkey were Dr. Kadriye Bilge Toprak, Dr. Rüçhan Kutbay and Dr. Emel Çobanoğlu. These pioneering Turkish women will always be remembered with respect.

As tremendous advances were happening in the country at this period, in 1951, at Gülhane Military Medical Academy, the first anaesthesia practices were started by Dr. Ali Ulvi Kaya and Dr. İsmail Bağcılar.^15^ In the following years, with the opening of new hospitals, new anaesthesia departments have been established. In Ankara Training Hospital in 1953, Dr. Hüsrev Polat started anaesthesia practices and, Dr Ulvi Kaya (1963), Dr. Emel Çobanoğlu and Dr. Turhan Candan contributed to the clinic.

A year later, in Training Regulation, dated 20^th^ January 1956 and declared in the 9212^th^ issue of Official Newspaper, numbered 4/6379, anaesthesiology was listed as a separate training field. Thus, the pioneers of this field started officially receiving their degrees.^12,13,14,15^ First two to be named are Dr. Sadi Sun, March 1956, from Istanbul University Medical School, and Dr. Cemalettin Öner, December 1956, from the Ministry of Health. Afterwards, in 1957, Dr. Moiz Kan, Dr. Cahit Bergil, Dr. Emel Berkol, Dr. Mehmet Nazlı, Dr Refik Paykoç, and Dr. Müfit Erkul, respectively; in 1958, Dr. Kamil Ergin, Dr. Faruk Or, and Dr. Orhan Toydemir were among the first ones to qualify to receive anaesthesia specialist degrees.^12,13,14,15^

In the 1950s, society of anaesthesologists was established in some countries. At the same period, anaesthesiology specialists Dr Sadi Sun, Dr Sabahat Kabaalioglu and Dr. Cezmi Kınoğlu came together with Dr. Şinasi Hakkı Erel and Dr. Fahri Arel to establish “*The Society of Anesthesia*” in 1956.^4,5,6,7,8,9,10,11,12,13,14,15^ The first president of the society was Dr. Fahri Arel. Sadi Sun was elected president on April 7, 1958. Its first congress was held in Istanbul on May 9, 1965. Later, this society will be named as “*The Society of Anesthesia and Reanimation*” (TARC) in 1969. Sixteen years after the establishment of the association, “*The Journal of Turkish Anesthesiology and Reanimation Society*” (1972) began to be published. With the decision of the Council of Ministers in 1975, the name “Turkish” would be officially added to the society.

In the developing and ever-changing Turkish Republic in 1958, the first independent “Anesthesiology Institute” under Ankara University Medical School. Ankara University was founded in 1948. Dr. Hilmi Akın, a surgeon, first led the administration since trained anaesthesia specialists were scarce. Afterwards, anaesthesiology specialist Dr. Refik Paykoç took over and improved the clinic.

After establishing period, Dr. Birsen Saygın who was the first female professor of anaesthesiology developed the clinic further (1994). Dr. Melek Tulunay in the intensive care, Dr. Yeşim Ateş in algology are important names in our history with their works from this clinic. In later years, Dr. Filiz Tüzüner developed the clinic further (1994).

In 1958, at Hacettepe University Medical School in Ankara, under the ANDAY section, which is the first nucleus of this university, Dr. Emel Çobanoğlu founded Anaesthesiology Department in 1963, Dr. Suat Karasu was assigned as the department chief. When Dr. Karasu resigned, Dr. Özdemir Demir, who returned to Ankara in 1964, was appointed to be his successor in 1965. In 1967, following the foundation of Hacettepe University Medical School, Dr. Özdemir Demir, Dr. Mualla Karamehmetoglu, who received her degree in 1966, and Dr. Kemal Erdem performed successfully as chief of the anaesthesiology and reanimation department.^8,9,12,13^  Later, Dr. Ülkü Aypar than Dr. Meral Kanbak will continue this duty successfully until their retirement.

While 1960 Turkish coup d’état was happened, Dr. Sadi Sun was named the first Associate Professor of Anaesthesiology in Turkey at Istanbul University Medical School.^7,8,9,10,11,12,13^ The same clinic, in 1961, was renamed as Anaesthesiology and Reanimation Institute.

While the intensive care is celebrating its 60^th^ anniversary this year, The concept as born from the devastating Copenhagen polio epidemic. Bjorn Ibsen, the anaesthesiologist who had suggested that positive pressure ventilation should be the treatment of choice during the epidemic, had set up the first intensive care unit in 1953. During this period, a remarkable development took place in Turkey and with the efforts of Dr. Cemalettin Öner, “Reanimation” was added to the anaesthesia title in 1963 (9-14). In the Education Regulation No. 6/821 of the Ministry of Health, the name of this scientific and hard-working physician specialty group was changed to “Anaesthesiology and Reanimation”.^11,13,15^

In 1960s, the Turkish economy grew at the expected target rate and, this constituted almost an industrial revolution and a take-off of a kind which few other world states had yet managed. Parallel to these developments, the anaesthesiology and reanimation clinics and departments were opened in every corner of the country. To name the pioneer physicians who founded anaesthesiology and reanimation clinics and departments and took part in many important developments: Dr. Cahit Bergil (1956), followed by Dr. Raife Torun (1966) in Şişli Etfal Training and Research Hospital (TRH); Dr. Bernard and Dr. Mustafa Dengilioğlu as first chief of clinic (1961), and followed by Nurten Ünal in Ankara TRH; Dr. Mehmet Ali Carfi, Dr. Semiramis Oyman (1958) in İzmir Katip Çelebi Atatürk TRH; Dr. Çetin Tuna and Dr. Bekir Mutlu as first chief of clinic (1972), and followed by Dr. Mehmet Yıldırım in Ankara Numune TRH; Dr. Ali Beşirikli (1961) in Adana Numune TRH; Dr. Ercüment Kopman (1970) and followed by Dr. Sevim Canik (1986), and Dr. Zuhal Aykaç (1987) in Siyami Ersek TRH; Dr. Ulvi Kaya (1961) and Dr. Turhan Candan as first chief of clinic (1970) in Dışkapı Yıldırım Beyazıt TRH; Dr. Hale Akoğuz, Dr. Faruk Müftüoğlu (1964), Dr. Çiğdem Yakut and Dr. Sevim Erbil (1980) in Turkish Yüksek İhtisas (High Training) TRH; Dr. Yıldız Köse (1969), followed by Dr. Sabahattin Uslu in Atatürk University Medical School; Dr. Ahmet Tutan (1957/1968), followed by Dr. İbrahim Yegül (1994), and Dr. Ali Reşat Moral (2000) in Ege University Medical School; Dr. Şevket Kaya (1969) and Dr. Mehmet Sarıbay as first chief of clinic (1974) in Taksim TRH; Dr. Ali Eren (1971) in Dicle University Medical School; Dr. Hale Akoğuz (1972), Dr. Hasan Akman, Dr. Gültekin Akoğuz, Dr. A. Geylan Işık, Dr. Uğur Oral, Dr. Dilek Özcengiz in Çukurova University Medical School; Dr. Orhan Toydemir (1974), followed by Dr. Gürayten Özyurt (1982), Dr. Oya Kutlay (1991), Dr. Gülsen Korfalı, and Dr. Şükran Şahin in Uludağ University Medical School; Dr. Şahin Yardım (1975) in Erciyes University Medical School; Dr. Zafer Pamukçu and Dr. Belkıs Tanrıverdi (1974) in Eskişehir Osmangazi University; Dr. Zeynep Esener (Kayhan) (1978), followed by Dr. H. Ayla Tür, Dr. B. Binnur Sarıhasan, and Dr. A. Haydar Şahinoğlu in Ondokuz Mayıs University.^13,15^

Despite to the political and economic problems during the 1970s in Turkey; Dr. Cemalettin Öner, Dr. Sadi Sun, Dr. Seyhan Çelikoğlu, Dr. Cemil Barlas, Dr. Umur Kaya, Dr. Hüsamettin Kerim Gökay, Dr. Faruk Or, Dr. Abdülkadir Erengül, Dr. Yıldız Köse, Dr. Beyhan Özden, Dr. Göksel Kalaycı, Dr. Kutay Akpir, Dr. Edip Kürklü, Dr. Dikmen Dolar and, Dr. Tuğrul Denkel established the “*Society of the Intensive Treatment and Care*” in 1978.

In the coming years, Gülhane Military Medical Academy has been consisting of two parts: Gülhane Military Medical Faculty (GATA) and GATA Haydarpaşa Training Hospital (1980). In GATA department of anaesthesiology; Dr. Nurettin Bayhan, Dr. Hikmet Süer, Dr. M. Erdal Güzeldemir, Dr. Ercan Kurt, Dr. Ahmet Coşar; in GATA Haydarpaşa training hospital Dr. Uğur Oral, Dr. Merih Gökben, Dr. Güner Dağlı and Dr. Sezai Özkan have been providing great contributions to the institution. Unfortunately, this well-established institution, whose roots date back to 1827, has been closed in 2016.

In addition to political problems in the 1980s, new economic development strategies were formulated, and the market economy was expanded. In historical process, many departments and clinics continued to be established in Republic of Turkey. Department of Anaesthesiology and Reanimation has been established and developed by Dr. Yener Karadenizli, Hülya Çelebi (1980), than Dr. Şahin Yardım in Gazi University; Dr. Nuri Erol İçel (1980) in Akdeniz University; Dr. Emel Sağıroğlu (1979) in Dokuz Eylül University; Dr. Safiye Atalay (1982) Karadeniz Teknik University; Dr. Osman Şengönül (1982) in Trakya University; Dr. Şeref Otelcioğlu (1983) in Necmettin Erbakan University; Dr. Güray Barlas (1983), followed by F. Yılmaz Göğüş (1986) in Marmara University Medical School; Dr. Nesrin Ertunç (1984) in Dr. Abdurrahman Yurtaslan Oncology TRH; Dr. Eser Şavkılıoğlu (1986) in Atatürk Pumonology and Thoracic Surgery TRH; Dr. Ünsal Öner (1990) in Gaziantep University.^6,8,15^

Anaesthesiology and reanimation specialists, effectively covering many medical areas, also took part in the development of algology. In 1986, the first algology clinic has been founded by Dr. Kadriye Bilge, and Dr. Serdar Erdine in İstanbul University School of Medicine. Society of Algology was founded in 1987 by Dr. Cemalettin Öner, Dr. Kutay Akpir, Dr. Kadriye Bilge ve Dr. Serdar Erdine as an anaesthesiologist. In 1990, first department of Algology was established by Dr. Serdar Erdine, and he made great contributions to the initiation and development of the science of algology in our country and the world. In 1990, Algology unit in İstanbul University İstanbul Medical Faculty was accepted as the first algology department in Turkey and contributed immensely to algology’s growth in our country and in the world. In these years, healthcare services have spread rapidly all over the world. Anaesthesiology and reanimation departments continued to be established in many university hospitals and training hospitals; Dr. Zuhal Arıkan (1987) in Kartal TRH; Dr. Ömer Lütfi Erhan (1987) in Fırat University Medical School; Dr. H. Aysel Altan (1988) in Okmeydanı TRH; Dr. Gülsen Bican (1988) in Haseki TRH; Dr. Sabahattin Uslu (1990) and Dr. Ayşe Gürel (1991) in İnönü University Medical School; Dr. Rıza Dediler (1993) in Yüzüncü Yıl University; Dr. Habip Atalay (1994) and Dr. Mustafa Gönüllü (1995) in Pamukkale University; Dr. Ahmet Tutan (1994) and Dr. Nurettin Lüleci (1994) in Manisa Celal Bayar University; Dr. Kamil Toker (1995) in Kocaeli University; Dr. Ali Dolgun (1996) in Süleyman Demirel University; and Dr. Uğur Oral (1999) in Mersin University.^13,15^ In the following years, university medical schools and training and research hospitals increased, making it possible to train new anaesthesiology and reanimation specialists who served nationwide.

In the 2000s, while the industry revolution 4.0 was started with internet and cyber-physical systems, the anaesthesiology and reanimation field reached high levels of education and service following its foundation’s first period in all over the world. During these years, the number of trainees receiving a high level of education has increased. Thus, on 29^th^ October 2001, Turkish Board of Anesthesiology and Reanimation (TARB) were founded to standardise clinical applications and supervise. The first board members were Dr. Kutay Akbir, Dr. Zeynep Kayhan, Dr. Aydemir Yalman, Dr. Özcan Erdemli, Dr. Serdar Erdine, Dr. Yüksel Keçik, Dr. Gülsen Korfalı, Dr. Mois Bahar, Dr. M. Erdal Güzeldemir and Dr. Hülya Çelebi.^11,15^ Since its establishment, TARB has being significant efforts in order to ensure highest level of training of anaesthesiology and reanimation residents and specialists for high quality and safe healthcare to all citizens.

During Republic’s history, in our country, following the formation of a scientific foundation of anaesthesiology specialty, anaesthesiology and reanimation specialists continuously provided care in operating room, intensive care units and pain clinics. When the date came to 2011, some radical changes began in our country to the detriment of anaesthesiology and reanimation specialists, and algology has been taken as a subspeciality program.

In 2012, intensive care was established as a subspeciality  program which caused many anaesthesiologists that worked in intensive care units to lose their rights as specialists. But now, more than six thousand anaesthesiologists with or without subspeciality degree with national consciousness and patriotism continue to serve in operating room, intensive care and pain clinics with excellent performance.

During the 2000s, following the improvement of state universities that keep increasing numbers, anaesthesiology and reanimation departments were founded in foundation universities, and new generation government universities. Dr. Gülnaz Aslan (1994) at Başkent University; Dr. Bora Aykaç (2005) at Yeditepe University; Dr. Levent Kılıkan, Dr. Refik Paykoç, Dr. Birsen Saygın (2006) at İstanbul Bilim University; Dr. Nigar Baykan (2008) in Acıbadem University, Dr. Ömür Erçelen (2010) in Koç University; Dr. Erdoğan Öztürk (2010) in Bezm-i Alem Vakıf University; Dr. Melek Güra Çelik (2012) in Medeniyet University; Dr. Hüseyin Öz (2012) in Medipol University; Dr. Osman Ekinci (2015) in Health Sciences University; Dr. Güner Dağlı (2017) in Sanko University are to name as department founders.^15^

Until now, biggest society of the anaesthesiology and reanimation specialists is the Turkish Society of Anesthesiology and Reanimation in Turkey, and continue working on educational activities, law and personal rights. Since the foundation of the Turkish Society of Anesthesiology and Reanimation (TARD), Dr. Sadi Sun, Dr. Bora Aykaç, Dr. Kutay Akpir, Dr. Uğur Oral, Dr. Oya Kutlay, Dr. Filiz Tüzüner, Dr. Mois Bahar, Dr. Ali Reşat Moral, Dr. Ülkü Aypar, Dr. Şükran Şahin, Dr. Güner Kaya, Dr. Neslihan Alkış, Dr. Hülya Bilgin, Dr. Ömer Kurtipek, Dr. Meral Kanbak, and Dr. Ali Fuat Erdem have been presidents of the TARD successfully in first 100 years of society respectively.

In 1972, 16 years later the establishment of the society, Turkish Journal of Anaesthesiology and Reanimation (TJARD) has been published. In the early years of the journal, between 1972-1985, Dr. Abdulkadir Erengül undertook the preparation task of the journal, which consists of the proceedings of the congress held the previous year, conference contents and some articles. In 1985, Dr. Sadi Sun assigned the editor-in-chief of the journal to Dr. Mois Bahar. Between 1985 to 2002, during his 17 years of duty; the journal began to be published 4 times, then 6, 8 and 10 times a year. Later Dr. Oya Kutlay, Dr. Filiz Tüzüner, Dr. Erdal Güzeldemir, then Dr. Melek Tulunay took over this duty with the high scientific and organizational performance. Dr. Yalım Dikmen, Dr. Nüzhet Mert Şentürk, and Dr. Aslı Dönmez have reached a high scientific level over the years. Today, TJARD is a very well-known journal which has a high scientific quality and indexed by national and international databases.

In addition to Turkish Society of Anesthesiology and Reanimation, Turkish Society of Intensive Care, Turkish Society of Algology, Anesthesiology and Reanimation Specialists’ Society (ARUD), Society of Thoracic and Cardiovascular Anesthesiology and Intensive Care, Society of Regional Anesthesia, Society of Clinical Enteral and Parenteral Nutrition (KEPAN), Turkish Resuscitation Council, Society of Palliative Care, Society of Clinical Toxicology were also founded by anaesthesiology and reanimation specialists.

While the date 2023, approximately 6000 anaesthesia and reanimation specialists are providing high quality healthcare in every corner of Turkey, sustaining education for physicians, and represents their country with the high scientific level internationally. Despite it is certain that they have a serious loss of rights in algology and intensive care subspecialties in recent years, anaesthesiology and reanimation specialists has continued to serve devotedly in these areas also in extraordinary circumstances such as pandemics and earthquakes.

## Conclusion

From the foundation of our Republic to this day, hundred years passed; everything started with pioneer anaesthesiology and reanimation specialists who worked relentlessly to build Republican Turkey and today, the torch is passed on to the anaesthesiology and reanimation specialists who still work as relentlessly as our pioneers. National data shows us that in 2022, the number of active hospitals will be 1563, a combination of 908 county hospitals affiliated with the Ministry of Health, foundation university hospitals, and 655 private hospitals. By the end of 2022, 5687 anaesthesiology and reanimation specialists and 3907 trainees will be actively working. In this case, it is expected that the number of anaesthesiology and reanimation specialists working in our country in the next 10 years will be around 15-20 thousand. This high number requires greater preparations in terms of maintaining the currently existing high standard of education and superior service quality. It may also bring about the problem of creating employment in the field of work; Precautions should be taken to prevent “a larger workforce that will work longer hours at lower wages”.

The increasing world and country population has brought about the need for more healthcare services. In parallel with this situation, it is necessary to take part in the dizzying changes in the field of technology in our country and the new digital revolution we are in. For us to be able to produce projects that will have a say in the world in the coming centuries, it is necessary to create different areas of expertise in our country with this awareness. In this process, in the rapidly changing world with technology and digitalization; also need to prepare our colleagues against situations such as crisis and depression that can lead to loneliness and inefficiency. The strength we need to keep providing a high level of education and for the next Republican generations to improve scientifically; is present “in the noble blood running through our veins”. Only with fair leaders, who can create a consciousness that brings out “trust and respect” in public for the physicians trained by us, can we successfully carry anaesthesiology and reanimation science forward for years to come.
